# The Specialized Roles in Carotenogenesis and Apocarotenogenesis of the Phytoene Synthase Gene Family in Saffron

**DOI:** 10.3389/fpls.2019.00249

**Published:** 2019-03-04

**Authors:** Oussama Ahrazem, Gianfranco Diretto, Javier Argandoña Picazo, Alessia Fiore, Ángela Rubio-Moraga, Carlos Rial, Rosa M. Varela, Francisco A. Macías, Raquel Castillo, Elena Romano, Lourdes Gómez-Gómez

**Affiliations:** ^1^Departamento de Ciencia y Tecnología Agroforestal y Genética, Facultad de Farmacia, Instituto Botánico, Universidad de Castilla-La Mancha, Albacete, Spain; ^2^Italian National Agency for New Technologies, Energy, and Sustainable Development, Casaccia Research Centre, Rome, Italy; ^3^Allelopathy Group, Department of Organic Chemistry, Institute of Biomolecules (INBIO), School of Science, University of Cádiz, Cádiz, Spain; ^4^VITAB Laboratorios, Albacete, Spain; ^5^Department of Biology, University of Rome Tor Vergata, Rome, Italy

**Keywords:** apocarotenoids, carotenoids, phytoene synthase, activity, expression, mycorrhiza, root, stigmas

## Abstract

*Crocus sativus* stigmas are the main source of crocins, which are glucosylated apocarotenoids derived from zeaxanthin cleavage that give saffron its red color. Phytoene synthase (PSY) mediates the first committed step in carotenoid biosynthesis in plants. Four *PSY* genes encoding functional enzymes were isolated from saffron. All the proteins were localized in plastids, but the expression patterns of each gene, *CsPSY1a*, *CsPSY1b*, *CsPSY2*, and *CsPSY3*, in different saffron tissues and during the development of the stigma showed different tissue specialization. The *CsPSY2* transcript was primarily detected in the stigmas where it activates and stimulates the accumulation of crocins, while its expression was very low in other tissues. In contrast, *CsPSY1a* and *CsPSY1b* were mainly expressed in the leaves, but only *CsPSY1b* showed stress-light regulation. Interestingly, *CsPSY1b* showed differential expression of two alternative splice variants, which differ in the intron retention at their 5′ UTRs, resulting in a reduction in their expression levels. In addition, the *CsPSY1a* and *CsPSY1b* transcripts, together with the *CsPSY3* transcript, were induced in roots under different stress conditions. The *CsPSY3* expression was high in the root tip, and its expression was associated with mycorrhizal colonization and strigolactone production. CsPSY3 formed a separate branch to the stress-specific Poaceae homologs but was closely related to the dicot PSY3 enzymes.

## Introduction

Carotenoids are a large family of isoprenoid compounds that are widely distributed in plants in which they exert numerous functions. Carotenoids in the chloroplast participate in photosynthesis, contribute to photoprotection, and provide biosynthetic precursors for strigolactones and abscisic acid (ABA) biosynthesis, which mediate developmental signaling and stress responses (Walter et al., 2010). As secondary metabolites, they accumulate in chromoplasts, providing attractive colors and aroma precursors for pollination and seed dispersal (Li and Yuan, 2013). In addition, carotenoids and their cleavage products, the apocarotenoids, have important health benefits (Fiedor and Burda, 2014) and contribute to the nutritional quality of horticultural crops (Yuan et al., 2015).

The phytoene synthase (PSY) gene family in both dicots and monocots is one of the best studied in plant carotenogenesis, since these enzymes catalyze the committed step and, in many plants, the rate-limiting reaction of the carotenoid pathway (Burkhardt et al., 1997; Hirschberg, 2001; Yao et al., 2018). The number of PSY genes clearly differs among species and has significance for the function and modulation of carotenogenesis in different tissues and under different environmental and developmental conditions (Shumskaya and Wurtzel, 2013). In addition, it has been shown that the abundance and stability of the PSY proteins in Arabidopsis and sweet potato are post-transcriptionally regulated by Orange (Or), which belongs to the DNAJ chaperone protein group (Zhou et al., 2015; Park et al., 2016), and are also affected by the activity of Clp proteases, which play an important role in intraplastid proteolytic processes (Welsch et al., 2018). Arabidopsis and most other Brassicaceae have only one PSY gene (Arabidopsis Genome Initiative, 2000), while two or more PSY genes have been reported for other plants, including major food staples in the grasses and other crops of agronomic and horticultural importance (Dibari et al., 2012).

Monocots contain two or more *PSY* genes. In maize, *PSY1* and *PSY2* are both strongly expressed in the leaves, and *PSY1* is more strongly expressed in yellow maize kernels than *PSY2* (Li et al., 2008b), while the transcript level of *PSY3* is associated with abiotic stress-induced carotenogenesis that produces ABA and is likely to be mediated by Or (Li et al., 2008a). Similarly, in rice, *PSY1* and *PSY2* are involved in carotenoid biosynthesis in green tissues, and *PSY3* is also up-regulated under different stress conditions (Welsch et al., 2008). In wheat also three *PSY* genes have been identified; the expression of *PSY1* expression was associated with β-carotene synthesis in the grain, while *PSY3* was highly expressed in the stem, leaves, seed developmental stages and under different stress conditions (Flowerika et al., 2016). Of the three PSY genes isolated from banana, two are related to *PSY2* of *Z. mays* and one to *PSY1* (Kaur et al., 2017). Finally, two *PSY* genes have been identified in the flowers of *Crocus ancyrensis*, with *PSY2* more strongly expressed than *PSY1*, and mainly associated with apocarotenoid biosynthesis in flower tissues (Ahrazem et al., 2015a).

Saffron is a well-known spice, whose color and aroma are due to the presence of the water-soluble apocarotenoids crocins, which confer a red bright coloration to the stigmas of *Crocus sativus*, and to volatile safranal, respectively (Ahrazem et al., 2015b). In addition to providing attractive flavor and coloration, which represent the primary factors for the quality of the spice, crocins and safranal are beneficial health compounds (Christodoulou et al., 2015). In *C. sativus* stigmas, crocins concentrations increase during the early developmental stages, reaching the highest concentrations in immature red stigmas (Moraga et al., 2009). We previously characterized the saffron apocarotenoid pathway and found a strong correlation between crocins accumulation in the stigmas and the transcript levels of three genes, β-carotene hydroxylase (*BCH*) (Castillo et al., 2005), lycopene-β-cyclase (*LCYB*) (Ahrazem et al., 2010), and carotenoid cleavage dioxygenase 2 (*CCD2*) (Rubio et al., 2008; Ahrazem et al., 2016). However, little is known about the saffron PSY, which plays a key role in the carotenoid pathway, since it is the enzyme that catalyzes the first committed step. In this study, we characterized the saffron *PSY* family, which is comprised of four members, and we investigated their roles by examining their overlapping or specialized roles during the development of the stigma and in other tissues, protein localization, enzymatic function, transcript levels and relation with the biosynthesis of the saffron apocarotenoids.

## Materials and Methods

### Plant Material and Treatments

*Crocus sativus* corms, donated by Fundación Valeriano González (Albacete, Spain), were used throughout the experiments. The corms were placed on pots and in the fields of Jardín Botánico de Castilla-la Mancha (Albacete, Spain). Leaves, roots, corms, and flowers were collected at the developmental stages previously described (Rubio et al., 2008; Lopez and Gomez-Gomez, 2009), frozen in liquid nitrogen and stored at −80°C until further use.

Ten saffron corms arranged two by two in pots and covered with soil were used for each different stress treatment. NaCl (Fluka) was dissolved in water and applied to the soil. Drought was induced by ceasing the supply of water to the plants.

To illuminate the dark-adapted leaves, five saffron plants were placed in dark chambers followed by illumination with 200 μmol m^−2^ s^−1^ for 4 h.

Mycorrhizal infection was performed as previously described (Shuab et al., 2016). Roots from the saffron plants were stained to detect mycorrhizae as previously described (Phillips and Hayman, 1970). The fresh roots from five plants were cut into 3 cm segments and cleared with 10% (w/v) KOH at 98°C for 30 min and rinsed in water three times. Next, the root segments were soaked in 0.1 N HCl for 1 min and stained overnight with 0.05% trypan blue. Four stained segments were mounted on one slide, and a total of 20 root segments from each root were examined under the microscope.

### Isolation of cDNA Sequences Encoding Phytoene Synthase Enzymes

*Crocus sativus* stigmas and tepals were used for total RNA extraction using an RNeasy Plant Mini Kit following the manufacturer’s instructions (Qiagen, Hilden, Germany). First-strand cDNAs were synthesized by reverse transcription (RT) from 1 μg of total RNA using an oligo dT primer and a First-strand cDNA Synthesis Kit (GE Healthcare Life Sciences, Buckinghamshire, United Kingdom) according to the manufacturer’s instructions. The cDNAs obtained were used as templates for degenerate PCR primers designed from the conserved motifs of the Crocus *PSY* genes (Moraga et al., 2009; Ahrazem et al., 2015a; Supplementary Table S1). The conditions for RT were as follows: 65°C for 5 min, 37°C for 1 h, and 75°C for 5 min. The thermal cycling parameters were 2 min at 95°C, 35× (30 s at 95°C, 20 s at 60°C and 1 min at 72°C) and finally 5 min at 72°C. The full-length clones were obtained using a RT-polymerase chain reaction of the 3′ and 5′ amplification ends (SMARTer^TM^ RACE cDNA Amplification Kit, Clontech, Palo Alto, CA, United States) using RNA from the stigma tissue and several primer combinations (Supplementary Table S1). The PCR products were separated in 1.0% agarose gels stained with ethidium bromide, purified, ligated into the pGEMT-easy vector (Promega, Madison, WI, United States) and introduced into *E. coli* cells.

### Cellular Localization

The C-termini of CsPSY1a, CsPSY1a, CsPSY2, and CsPSY3 were fused to the N terminus of eGFP in the pBI-eGFP vector (Shi et al., 2005) using an In-Fusion^®^ HD Cloning Plus CE kit (Clontech ^1^) and specific primers (Supplementary Table S1). The four constructs were examined by sequencing before transformation in *Agrobacterium tumefaciens* strain C58C1. The transient expression of the *CsPSY* genes has been performed by agroinfiltration of *Nicotiana benthamiana* leaves as previously described (Frusciante et al., 2014). After 5–7 days postinfection, the leaves were analyzed using a confocal laser scanning microscope as previously reported (Frusciante et al., 2014). Lasers 488 nm (argon) and 635 nm (diode) were used to detect the eGFP (green, 488 nm/505–540 nm) and chlorophyll (red) fluorescence, respectively, with a 505 to 540 nm band-pass emission filter for eGFP, and 660 to 750 nm emission wavelengths for chlorophyll auto-fluorescence. Images of 800 × 800 pixels were acquired in xyz scan mode using a 60× objective (numerical aperture 1.35) with optical zooming 3× and further analyzed with IMARIS (Bitplane) software.

### Phylogenetic Analysis

The amino acid sequences were aligned using the BLOSUM62 matrix with the ClustalW^2^ algorithm-based AlignX module from MEGA Version 7.0^3^ (Tamura et al., 2013), and used to generate a Neighbor-Joining tree with bootstrap support (2,500 replicates). Gaps were deleted pairwise.

### Isolation of Genomic Clones

Genomic DNA was prepared from *C. sativus* leaves using an i-Genomic Plant DNA Extraction Kit (iNtRON Biotechnology, Sangdaewon-Dong, South Korea) and used to isolate gene sequences using specific oligonucleotides (Supplementary Table S1). All the PCR reactions were performed using an Advantage 2 Polymerase mix (BD Biosciences, Palo Alto, CA, United States). The reaction products were ligated to pGEM-T using a TA Cloning Kit (Promega Corporation, Madison, WI, United States). The ligated DNA was transformed into *E. coli*. Colonies were individually picked, amplified, and the plasmid DNA was extracted using a DNA Plasmid Miniprep Kit (Promega, Madison, WI, United States) for each amplification round. The plasmids were sequenced using an automated DNA sequencer (ABI PRISM 3730xl, PerkinElmer) from Macrogen Inc. (Seoul, South Korea).

### DNA Sequencing and Analysis of the DNA and Protein Sequences

The clones obtained were sequenced using an automated DNA sequencer (ABI PRISM 3730xl, PerkinElmer, Macrogen Inc. ^4^). Similarity searches were performed using the BLAST suite of programs of the National Center for Biotechnology Information (NCBI^5^). Motif searches were performed using PROSITE^6^, SignalP^7^, DeepLoc-1.0^8^, and TMpred^9^. The proteins were modeled using Swiss-mode and Swiss-PdbViewer^10^. Membrane interactions of the three-dimensional protein structures were constructed using PPM Server^11^. The entire 5′ UTR of *CsPSY1b* with or without intron structures and their minimum free energies (MFEs) were calculated using RNAfold (Lorenz et al., 2011) with default parameters.

### Expression Analysis

For expression analyses of PSY genes in stigmas, stamens and tepals, flowers were dissected from five plants and were designated as a sample, and biological replicates (in total five samples) were used for total RNA extraction. For expression analyses using leaf and root material, the tissues were independently collected from five plants, and these five biological replicas were used for total RNA extraction analyses. In the case of the tissues collected for the stress experiments, the roots from each of the pots subjected to a certain treatment were dissected and were designated as a sample (in total five samples for each treatment). For all the samples, RNA was extracted using an RNeasy Plant Mini Kit following the manufacturer’s instructions (Qiagen, Hilden, Germany). First-strand cDNAs were synthesized by RT from 1 μg of total RNA using an oligo dT primer and a First-strand cDNA Synthesis Kit (GE Healthcare Life Sciences, Buckinghamshire, United Kingdom) according to the manufacturer’s instructions. The cDNAs obtained were used as templates using gene-specific RT-qPCR primers (Supplementary Table S1). Transcript levels of the *PSY* genes were normalized with those of *RPS18* (Rubio-Moraga et al., 2014), and each RNA sample was assayed in triplicate. The cycling parameters of qPCR consisted of an initial denaturation at 94°C for 5 min, 40 cycles at 94°C for 20 s, 58°C for 20 s, 72°C for 20 s, and a final extension at 72°C for 5 min. The assays were conducted in a StepOne^TM^ Thermal Cycler (Applied Biosystems, Foster City, CA, United States) and analyzed using StepOne software v2.0 (Applied Biosystems, Foster City, CA, United States). DNA melt curves were created for each primer combination to confirm the presence of a single product.

### Plasmids and Functional Complementation

pAC-85b was used to investigate the biological function of all four CsPSY proteins. The plasmid pAC-85b (Cunningham and Gantt, 2007) contains the *crtE*, *crtI*, and *crtY* genes from *Erwinia herbicola*, which encode the GGPP synthase, phytoene desaturase and lycopene cyclase enzymes, providing all the enzymes necessary to synthesize β-carotene, with the exception of PSY. The *E. coli* cells transformed with pAC-85b could not synthesize any carotenoid, which resulted in white bacterial colonies. The *CsPSY* genes were cloned separately into the *Eco*RI site of the pBAD-Thio vector (Invitrogen ^12^) by recombination using an In-Fusion^®^ HD Cloning Plus CE kit (Clontech, see text footnote^1^) and specific primers (Supplementary Table S1). Chemically competent BL21 *E. coli* cells harboring the pAC-85b plasmid were prepared and transformed with the pTHIO plasmids harboring the *CsPSY* genes and the pAtPSY plasmid harboring the *PSY* of Arabidopsis used as a positive control. Positive colonies were inoculated in 5 mL of 2× YT media containing the antibiotics ampicillin (50 μg/mL) and chloramphenicol (25 μg/mL) and grown overnight at 30°C at 190 rpm. The overnight cultures were used to inoculate 50 mL 2× YT and cultured at 30°C until an optical density of 0.8 at 600 nm (OD_600_) was reached. The cells were induced with 0.2% arabinose and grown overnight at 20°C. The cells were harvested by centrifugation (6,000 rpm for 10 min), and the pigments were repeatedly extracted with a total volume of 10 mL of acetone until the pellet was colorless. The solvent was evaporated under N_2_ gas, and the pigments were resuspended with 0.3 mL MeOH:tertmethylbutylether (50:50, v/v). After centrifugation (13,000 rpm for 10 min), the extracts were analyzed using HPLC as previously described (Castillo et al., 2005).

### Strigolactone Measurement and Determination

A total of 50 mg of ground roots, obtained from six plants, were extracted in an ultrasonic bath (Selectra Ultrasonics, Barcelona, Spain) with 1 mL of ethyl acetate for 10 min. The samples were centrifuged for 10 min at 5,000 rpm. The organic phase was carefully transferred to glass vials. This procedure was repeated three times. Finally, samples were concentrated in a Rotavapor and stored at −80°C. The extracts were dissolved with MeOH to achieve a ratio of 1:1 g/L. As internal standard (±)-GR24 was dissolved in MeOH to achieve a concentration of 10 mg/L, and this was added to all the samples at 10 μg/L.

The samples were analyzed on a Bruker EVOQ Triple Quadrupole Mass Spectrometer using as ionization source an electrospray (ESI) in the positive mode. The samples were injected and separated using an ACE Excel 1.7 C18 (100 mm × 2.1 mm, 1.7 μm particle size) (Advanced Chromatography Technologies Ltd., Aberdeen, Scotland) maintained at 40°C. The mobile phases were solvent A (water, 0.1% formic acid) and solvent B (MeOH, 0.1% formic acid), and the flow rate was set to 0.3 mL/min. The linear gradient system was as follows: 0–0.5 min, 50% B; 0.5–5 min, to 100% B; 5–7 min, 100% B; 7–7.5 min, to 50% B, and 7.5–10.5 min, 50% B. The autosampler was set at 5°C to preserve the samples. The injection volume was 5 μL. The instrument parameters were as follows: spray voltage +4500 V, cone temperature 300°C, cone gas flow 15 psi, heated probe temperature 400°C, heated probe gas flow 15 psi, nebulizer gas flow 55 psi and collision pressure 2.0 mTorr. The compound-dependent parameters for orobanchol and the IS, the parent or precursor ions, the fragments obtained by MRM analysis and the collision energy to achieve each fragmentation are provided in Supplementary Table S4. In addition, using the same conditions, the following strigolactones were also examined: 7-oxoorobanchyl acetate (LOD = 2.8 μg⋅L^−1^), solanacol (LOD = 19.2 μg⋅L^−1^), strigol (LOD = 5.2 μg⋅L^−1^), fabacyl acetate (LOD = 0.8 μg⋅L^−1^), orobanchyl acetate (LOD = 17.0 μg⋅L^−1^), and 5-deoxystrigol (LOD = 0.4 μg⋅L^−1^). However, none of them were found in the samples.

A stock standard solution of orobanchol at 10 mg/L was prepared in MeOH. An external standard calibration curve was prepared from a serial dilution of the working standard solution from 100 to 0.5 μg/L (9 levels, *R*^2^ = 0.9974). In addition, the IS (±)-GR24 was dissolved in MeOH to achieve a concentration of 10 mg/L, and this was added to all the samples at 10 μg/L. Orobanchol was supplied by Professor Xiaonan Xie and Professor Koichi Yoneyama (Weed Science Center, Utsunomiya University, Japan), and (±)-GR24 was provided by Professor Binne Zwanenburg (Department of Organic Chemistry, Radboud University, Nijmegen, Netherlands).

## Results

### Characterization of the PSY Protein Family in Saffron

**FIGURE 1:**
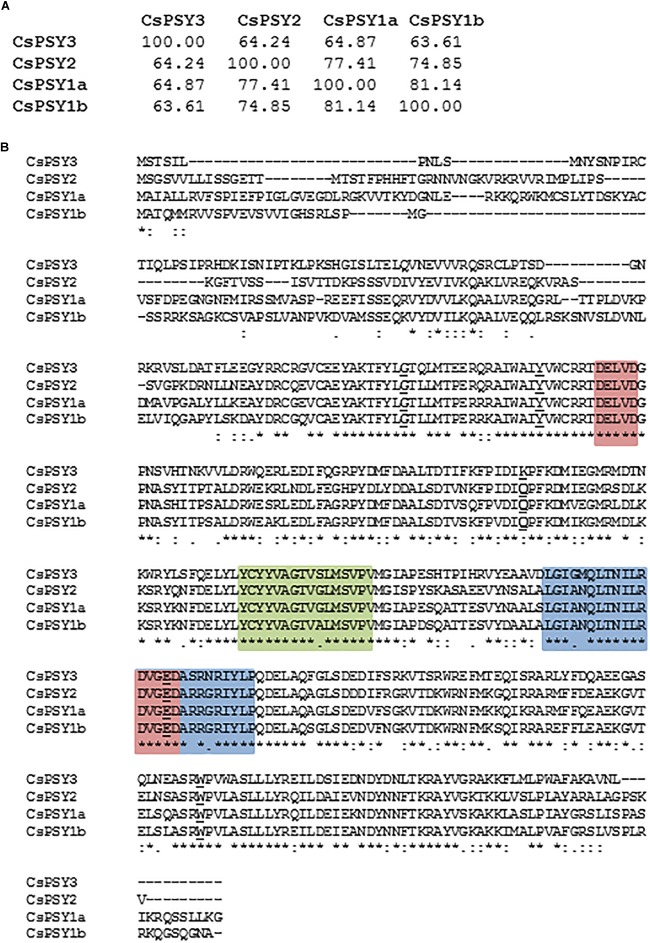
PSY amino acid sequence similarities. **(A)** Pairwise percentage similarity of the Crocus PSY mature amino acid sequences without the putative signal peptide. **(B)** Alignment of the amino acid sequences of the putative CsPSYs. With red background: aspartate rich regions and substrate-Mg2+-binding sites (DXXXD); black underline: active site lid residues. Green background: SQS-PSY domain 1; and blue background: SQS-PSY domain 2.

**FIGURE 2:**
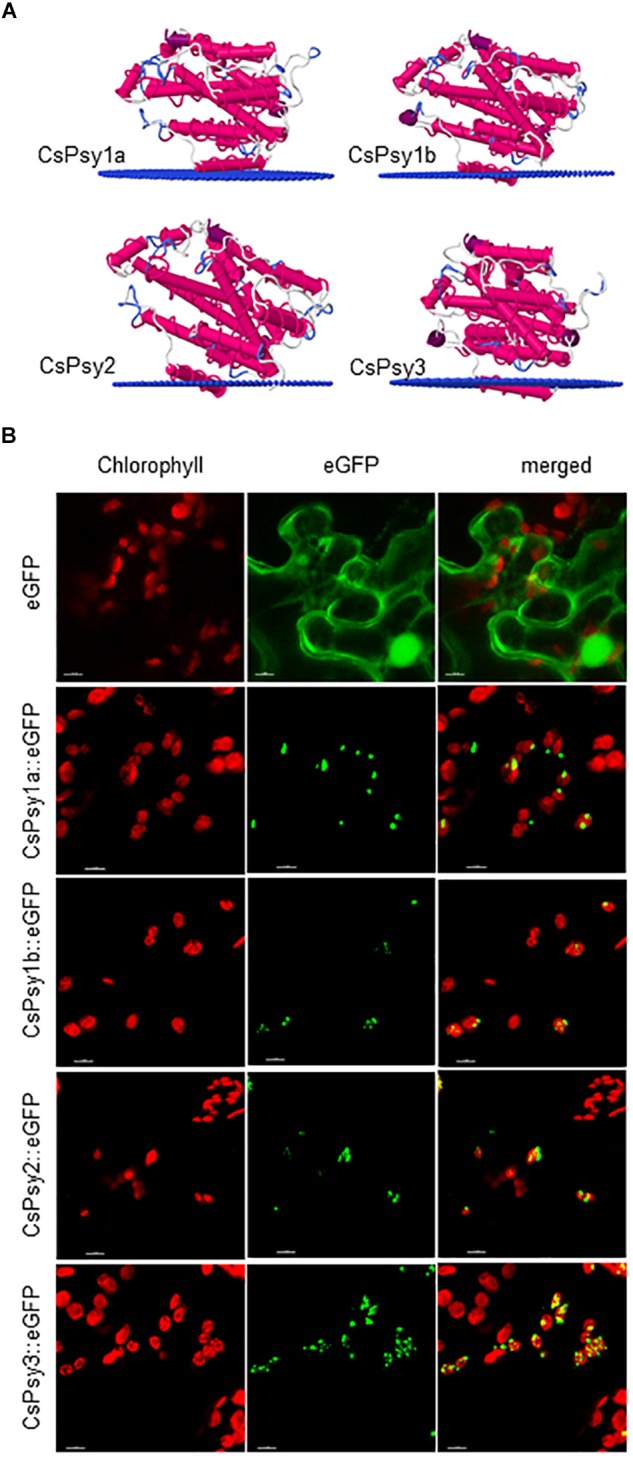
Three dimensional models and location of the saffron phytoene synthase enzymes. **(A)** Three-dimensional models of the CsPSY enzymes. The models for CsPSY1a, CsPSY1b, CsPSY2, and CsPSY3 were created using the PPM Server (http://opm.phar.umich.edu/server.php). The α-helices, and loops are depicted as pink and blue, respectively. Blue dots indicate the membrane surface. **(B)** Subcellular localization of GFP fusion proteins of CsPSY1a, CsPSY1b, CsPSY2, and CsPSY3 in agro-infiltrated tobacco leaves after 5 days as detected with confocal laser scanning microscopy and enhanced green fluorescent protein (eGFP) expression. Chlorophyll auto-fluorescence in red (left panel), eGFP fluorescence is shown in green (middle panel) and a merged overlay of the eGFP/chlorophyll fluorescence (right panel) is shown in yellow.

Previously, two *PSY* genes were identified in *C. ancyrensis* and *Crocus sieberi* flowers (Ahrazem et al., 2015a, 2018), and one partial clone in saffron (Moraga et al., 2009). Using a PCR approach based on the conserved domains and degenerative primers (Supplementary Table S1) in the Crocus *PSY* family, we identified four *PSY* paralogs in saffron: *CsPSY1a*, *CsPSY1b* and *CsPSY2* and *CsPSY3* (Supplementary Table S2) with all the CsPSYs exhibiting a similarity between 58 and 76% (Figure 1A). The main differences among the isolated PSY enzymes were observed at the N-terminus region (Figure 1B), which might be partially due to the plastid transit peptides, known for reduced sequence conservation. Therefore, we used TargetP and DeepLoc-1.0^13^ to identify putative subcellular localization domains in the PSY amino acid sequences and TMHMM Server v. 2.0^14^ to determine the presence of putative hydrophobic domains in the structure of the isolated enzymes, which were compared with those present in the ZmPSY enzymes (Supplementary Figure S1). The PSY plant enzymes are localized in plastids, mainly in the plastoglobuli, although the PSY1 enzymes from rice and maize displayed different suborganellar localization (Shumskaya et al., 2012). Previous results from a proteomic analyses on the saffron chromoplast from stigmas displayed the presence of several peptides with homologies to the PSY enzymes (Gomez-Gomez et al., 2017). Analyses of such peptides showed their presence in the sequences of CsPSY1a, CsPSY1b, and CsPSY2. *In silico* analyses of the PSY proteins from saffron predicted a plastid location for all the proteins tested (Supplementary Figure S1), and a hydrophobic region was predicted in the N-t region of CsPSY1a, as well as in CsPSY1b and CsPSY2, which is present in the rice PSY genes that are targeted to the plastoglobules (You et al., 2016; Supplementary Figure S1). However, this hydrophobic region was not present in CsPSY3, suggesting a different location inside the plastid. In addition, putative membrane-interacting domains were identified within the tridimensional structure of CsPSY1b, CsPSY2, and CsPSY3, but not for CsPSY1a (Figure 2A). The PSY enzymes from rice and maize have been shown to interact with the membranes (Li et al., 2008b; Welsch et al., 2008). The tridimensional structures were modeled using the C(30) carotenoid dehydrosqualene synthase crystal structure from *Staphylococcus aureus* as template with 100% confidence on the 72% coverage, and a 30% amino acid sequence identity (Liu et al., 2008).

*In silico* analysis of the deduced amino acid sequences from the cDNAs obtained showed the presence of four characteristic sites inside the trans-isoprenyl diphosphate synthase domain, present in phytoene synthases (Dogbo et al., 1988). This domain of PSY catalyzes the head to head (1′–1) condensation of two molecules of geranylgeranyl diphosphate (GGPP) to produce phytoene. The domain included a substrate-Mg^2+^-binding site (aspartate rich region), a substrate-binding pocket, catalytic residues and active site lid residues (Figure 1B). A conserved domain analysis revealed that all four CsPSY proteins belong to the class 1 superfamily of isoprenoid biosynthetic enzymes containing the conserved trans-isoprenyl diphosphate synthases, and the head-to-head (trans-IPPS_HH) domain. ScanProsite analysis showed the presence of squalene/phytoene synthase signatures 1 and 2 (SQS-PSY1 and 2) in all the CsPSY proteins with PSY activity. The SQS-PSY1 motif (consensus pattern: Y-[CSAM]-x(2)-[VSG]-A-[GSA]-[LIVAT]-[IV]-G-x(2)-[LMSC]-x(2)-[LIV]) was found in each CsPSY protein with the consensus sequence YCYYVAGTVgLMSVPV; and the SQS-PSY2 motif (consensus pattern: [LIVM]-G-x(3)-Q-x(2,3)-[ND]-[IFL]-x-[RE]-D-[LIVMFY]-x(2)-[DE]-x(4,7)-R-x-[FY]-x-P) was found in all the CsPSY sequences with the consensus sequence LGIanQLTNILRDVGEDArRgRIYLP (Figure 1B). In addition, the aspartate-rich motifs DELVD and DVGED were conserved in each CsPSY protein.

### All Four CsPSY Enzymes Are Located in Plastids

To ascertain the targeting of the CsPSYs, we fused PCR amplified fragments encoding each CsPSY, including their predicted transit peptides, to the green fluorescent protein (eGFP). The fusion constructs were transiently expressed in *N. benthamiana* leaves and analyzed using fluorescent confocal microscopy (Figure 2B). All the CsPSY::eGFP were localized with chlorophyll in the chloroplast confirming that they are translocated to the plastids (Figure 2B). None of the enzymes was distributed throughout the plastid suggesting that the CsPSYs were not soluble enzymes. CsPSY1b, CsPSY2, and CsPSY3 were localized to speckles associated with the chloroplasts (Figure 2B); these speckles have been identified as plastoglobuli (Rubio et al., 2008; Shumskaya et al., 2012). However, CsPSY1a showed a different distribution and was present in a larger and fewer inclusions inside the plastid, suggesting the occurrence of oligomerization events in specific sites within the plastids (Figure 2B; Sun et al., 2018).

### CsPSY3 Is Closely Related to the Dicot PSY3 Sequences but Separated From the Other PSY3 Monocot Sequences From the Poaceae Family

**FIGURE 3:**
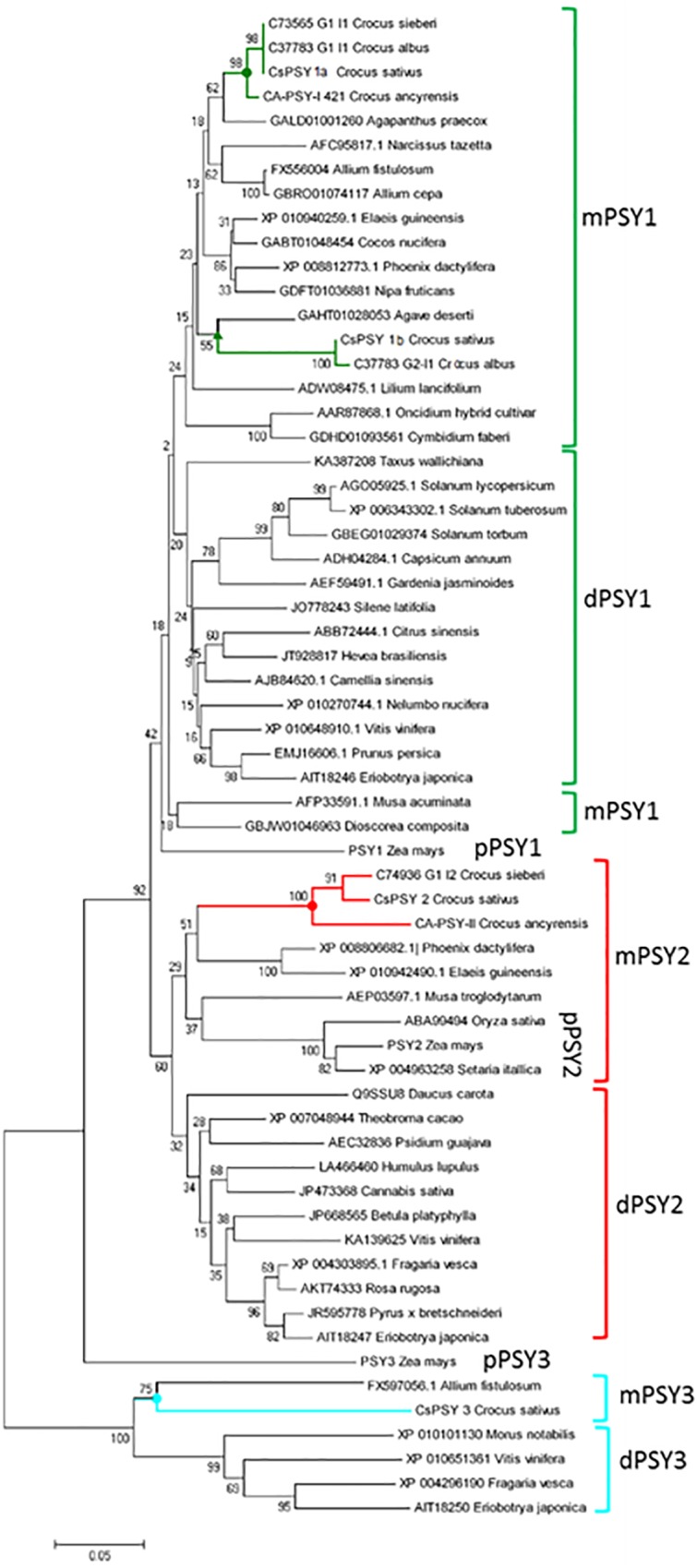
Dendrogram of the CsPSYs amino acid sequences with other plant members of the PSY family. The unrooted phylogenetic tree was constructed using MEGA7 from the PSY sequences retrieved from the GenBank database. Evolutionary relationships were inferred using the Neighbor-joining method with 2500 bootstrap re-sampling strategy. The PSYs sequences from Crocus species are indicated by different colors.

We performed phylogenetic relationships of the isolated enzymes with the PSYs from other plant species (Figure 3). The PSY proteins from maize (ZmPSY1, AAR08445; ZmPSY2, AAX13807; and ZmPSY3, DQ356430) were used as reference sequences to distinguish the different PSY types identified in monocots. The PSY phylogenetic tree revealed three main PSY subfamilies (Figure 3). The protein sequences of CsPSY1a, CsPSY1b, CsPSY2, and CsPSY3 group together with other monocot sequences. The phylogenetic analysis showed lineage-specific expansion and divergence for the Crocus sequences inside the PSY1 family (Figure 3). Orthologs for CsPSY1a were found in *C. cartwrightianus* Albus, *C. ancyrensis* and *C. sieberi*, but, non-orthologs were found in *C. ancyrensis* and *C. sieberi* for CsPSY1b (Ahrazem et al., 2015a, 2018). Interestingly, the non-poaceae monocot proteins inside the PSY1 and PSY3 clusters were more closely related to the dicot sequences than the PSY2 proteins. The CsPSY3 sequence, together with a PSY sequence form *Allium fistulosum* (FX597056.1), resulted in a more strong association to the PSY3 from dicotyledonous plants than from the ones isolated from the Poaceae family (Figure 3 and Supplementary Figure S2). The NCBI database was searched for PSY3 orthologs in other monocots, using the CsPSY3 sequence as bait, but only protein sequences inside the Liliopsida family were identified (Supplementary Figure S2), although several monocotyledonous genomes have been sequenced and annotated, such as the ones from *Musa acuminata* (Assembly accession GCF_000313855.1), *Elaeis guineensis* (Assembly accession GCA_000442705.1), *Phoenix dactylifera* (Assembly accession GCA_000413155.1), *Cocos nucifera* (GigaDB, RRID:SCR_004002), *Phalaenopsis equestris* (PRJNA192198), *Ananas comosus* (PRJNA371634), *Zostera marina* (PRJNA280336), and *Spirodela polyrhiza* (PRJNA308109). These results suggest the absence of PSY3-orthologs in these monocot species.

### Expression Analyses of the Isolated Genes in Several Tissues

**FIGURE 4:**
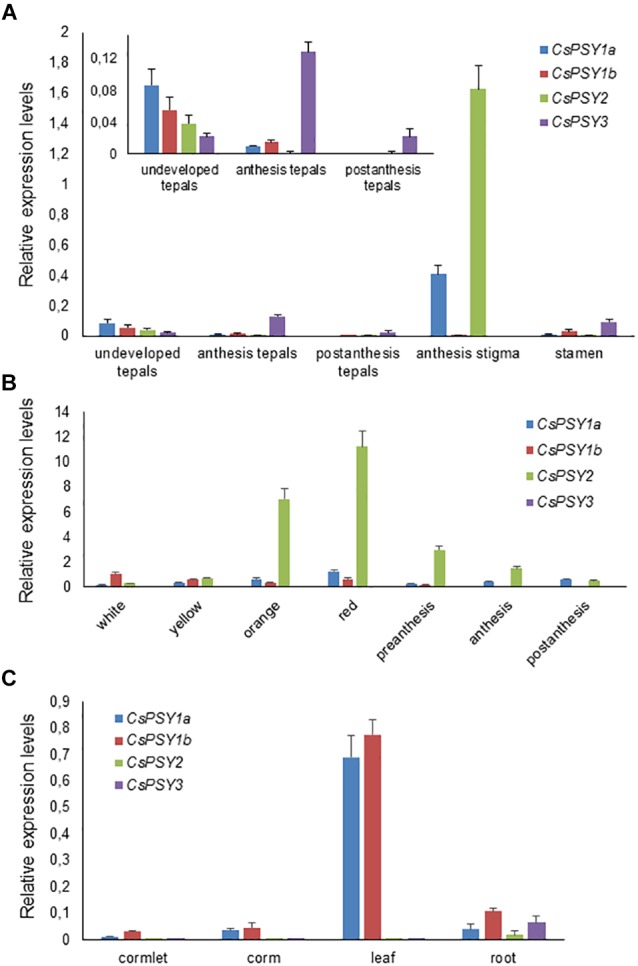
Relative expression levels of the *CsPSYs* in vegetative and reproductive tissues investigated by qRT-PCR. **(A)** Transcripts levels in the tepals at different developmental stages, and in the stigma and stamens at anthesis. **(B)** Expression levels in seven developmental stages of the stigma. **(C)** Transcripts levels in the cormlet, corm, leaf and roots. Scale bars indicate five biological replicates ± SD.

**FIGURE 5:**
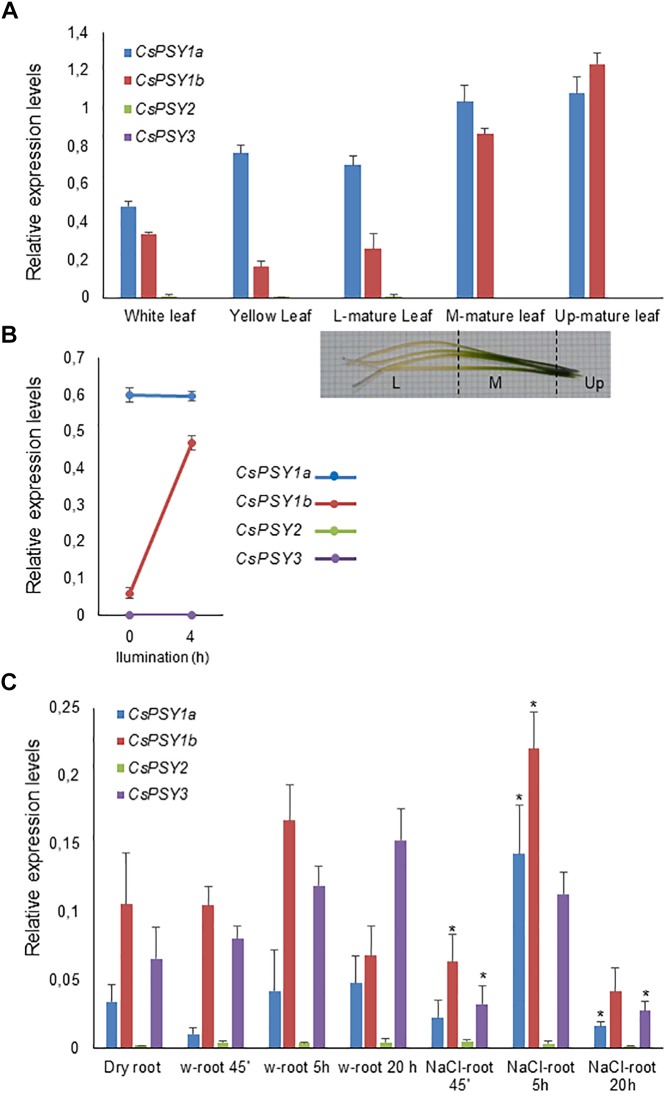
Stress regulation of the *CsPSYs* transcripts in leaf and root tissue. **(A)** qRT-PCR analysis of the *CsPSY* gene expression in leaves at different developmental stages and along the mature leaf (L, low part of the leaf; M, medium part of the leaf; Up, upper part of the leaf). **(B)** Transcript levels in leaves dark-adapted for 2 h and upon illumination with white light for 4 h. **(C)** Transcript levels of the *CsPSYs* genes in the roots of dry plants, re-watering plants and in plants treated with 200 mM NaCl. Error bars indicate five biological replicates ± SD. Asterisks mark statistically significant differences relative to reference (no NaCl addition) samples (*P* < 0.05).

To address the possibility of functional divergence among the *CsPSY* isoforms, we conducted a comprehensive characterization of their expression patterns in different tissues. Quantitative real time PCR was used to test the expression in the leaves, roots, corm, and flowers, and in the developing and mature tepals, stigmas and leaves. The results indicated the differential regulation of *CsPSY1a*, *CsPSY1b*, *CsPSY2*, and *CsPSY3* in the tissues analyzed (Figure 4, 5). All four *CsPSY* mRNAs were present in the floral tissues, although at very different levels (Figure 4A). *CsPSY2* was highly expressed in the stigma tissues (Figure 4A,B), reaching the highest expression levels in the red stage and decreasing afterward in the subsequent developmental stages. The expression level of *CsPSY2* was very low in the other tissues analyzed (Figure 4, 5), suggesting its involvement in the accumulation of crocins in the stigma tissue (Moraga et al., 2009). *CsPSY1a* showed a different pattern of expression during the development of the stigmas with higher levels of expression in the red stigmas, decreasing in preanthesis stigmas and again increasing its expression levels in anthesis and postanthesis stigmas (Figure 4B). Both *CsPSY1a* and *CsPSY1b* were mainly expressed in the leaves (Figure 4C) in all the developmental stages analyzed (Figure 5A), and their expression was higher in the completely green part of the leaf, suggesting that the CsPSY1a and CsPSY1b enzymes are mainly responsible for the carotenoid supply in the chloroplasts. However, some differences in the expression patterns in the leaves were observed between *CsPSY1a* and *CsPSY1b*. The *CsPSY1a* transcript levels were higher in the white and yellow immature leaves, and the *CsPSY1a* transcript levels were higher in the basal part of the mature leaves (L-mature leaf), which is not exposed to light, while the *CsPSY1b* levels increased in those mature leaves parts exposed to light (Figure 5A), suggesting a role for *CsPSY1b* in photoprotection. In addition, the effect of rapidly increased light intensity was tested with leaves that were dark-adapted for 2 h and illuminated with white light for 4 h (Figure 5B). A seven to eightfold induction of *CsPSY1b* was observed, while *CsPSY1a* was largely unresponsive (Figure 5B). The *CsPSY3* transcript levels were comparably low in all the tissues, but its levels were relatively high in the tepals at anthesis in addition to the stamens and roots (Figure 4A,C) and were undetectable in the stigma tissues.

### *CsPSY1a*, *CsPSY1b*, and *CsPSY3* Are Induced Under Stress Conditions

When growing saffron in the field, there is always interval occurrence in drought and/or rewetting events. Since a role in the stress response mechanisms has been previously reported for certain PSY members (Ruiz-Sola et al., 2014), saffron plants were grown in plastic pots and maintained with no water for 2 weeks to reproduce the field conditions of drought stress by treating only with distilled water or with distilled water supplemented with 250 mM NaCl. The time course of the accumulation of the *CsPSY* transcripts in the roots was measured during the first 45 min, 5 and 20 h after the treatments to evaluate the stress-induced expression in saffron PSY activities. Five hours after watering the plants, an increase in the expression levels of *CsPSY1b* was observed, followed by a marked decrease 20 h later. In contrast, the *CsPSY1a* transcript content was reduced 45 min after watering, and the expression levels returned to the original values 5 h later, remaining stable in the next time point (20 h) (Figure 5C). The *CsPSY3* transcript levels increased after 5 h in the roots and continued increasing 20 h later (Figure 5C). The *CsPSY1a*, *CsPSY1b*, and *CsPSY3* transcript levels were induced 5 h after salt treatment in the roots of the plants treated with NaCl (Figure 5C), and the expression of *CsPSY1a* and *CsPSY1b* were approximately two and fourfold higher than in the untreated-NaCl roots, respectively. However, the transcript levels of *CsPSY3* at this time point were similar to the ones observed in the first experiment, but 20 h after the salt treatment, the transcript levels of *CsPSY3* declined to values similar to the ones observed 45 min after the treatment. This reduction was also observed for *CsPSY1a* and *CsPSY1b* (Figure 5C), which also decreased in the water treatment.

### *CsPSY3* Expression Is Induced During Arbuscular Mycorrhizal Symbiosis in Saffron

**FIGURE 6:**
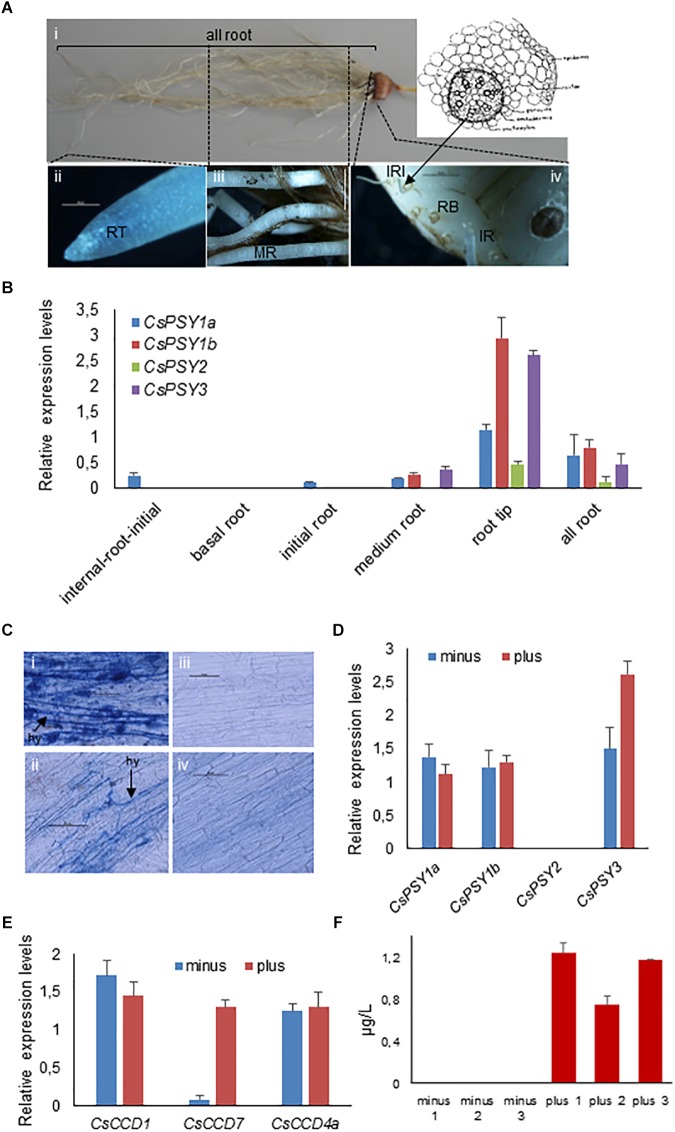
Expression analysis of the *CsPSY* genes in the root tissue. **(A)** The root system of saffron plants. i, the complete root system; ii, the root tip (RT); iii, the mature root (MR); iv, the corm showing the radicular bottom (RB), the initiation of the roots (IR), and the dissection of the IR, showing the root vascular cylinder (IRI). Bar = 50 μm. **(B)** qRT-PCR-based expression analysis of *CsPSY* genes in different parts of saffron roots. Expression levels are shown relative to the constitutively expressed RSP18 gene. Error bars indicate SD from three technical replicates. **(C)** Trypan blue staining of saffron roots with (i and ii) and without mycorrhization (iii and iv). Error bars = 50 μm. **(D)** qRT-PCR based expression analysis of the *CsPSY* genes in roots with or without mycorrhiza. **(E)** qRT-PCR based expression analysis of the *CsCCD* genes in roots with mycorrhiza or without. Expression levels are shown relative to the constitutively expressed saffron RSP18 gene. Error bars indicate SD from five biological replicates. **(F)** Orobanchol levels in roots with or without mycorrhiza. Error bars indicate SD from three biological replicates.

A more detailed analysis on the transcript levels of each *CsPSY* was conducted on the root tissues. Samples from saffron plants growing in pots were dissected in several parts (Figure 6A). For all the *CsPSYs* genes, the expression levels were higher in the root tip with *CsPSY1b*, followed by *CsPSY3*, showing the highest transcript levels. *CsPSY1b* was expressed in all the parts tested, with the exception of the basal root. In the medium root (mature root), *CsPSY3* showed the highest level of expression of all the four *CsPSY* transcripts analyzed (Figure 6B), while the *CsPSY1a* transcripts were the only ones detected in the root vascular cylinder (internal root initial) (Figure 6B).

The PSY3 enzymes from dicotyledonous plants are regulated by nutrient stress and mycorrhization (Stauder et al., 2018). Due to the closer identity of the CsPSY3 with the dicotyledonous PSY3 enzymes, an additional experiment was performed on cDNA samples prepared from root tissues with or without the presence of mycorrhizal fungi (Figure 6C; Phillips and Hayman, 1970). Only *CsPSY3* expression was strongly enhanced in the colonized roots (Figure 6D). In addition, the expression of *CsCCD7*, *CsCCD1*, and *CsCCD4a* in these samples (Figure 6E) showed the upregulation of the former (*CsCCD7*) in the mycorrhized roots. Due to the involvement of CCD7 in strigolactone biosynthesis and their role in the rhizosphere favoring mycorrhizal symbiosis establishment (Akiyama, 2007; Garcia-Garrido et al., 2009), the strigolactone content in colonized and non-colonized roots was analyzed and quantified using LC-MS/MS. Interestingly, the strigolactone orobanchol was only detected in the colonized roots (Figure 6F).

### Functional Analysis of All the CsPSYs

**FIGURE 7:**
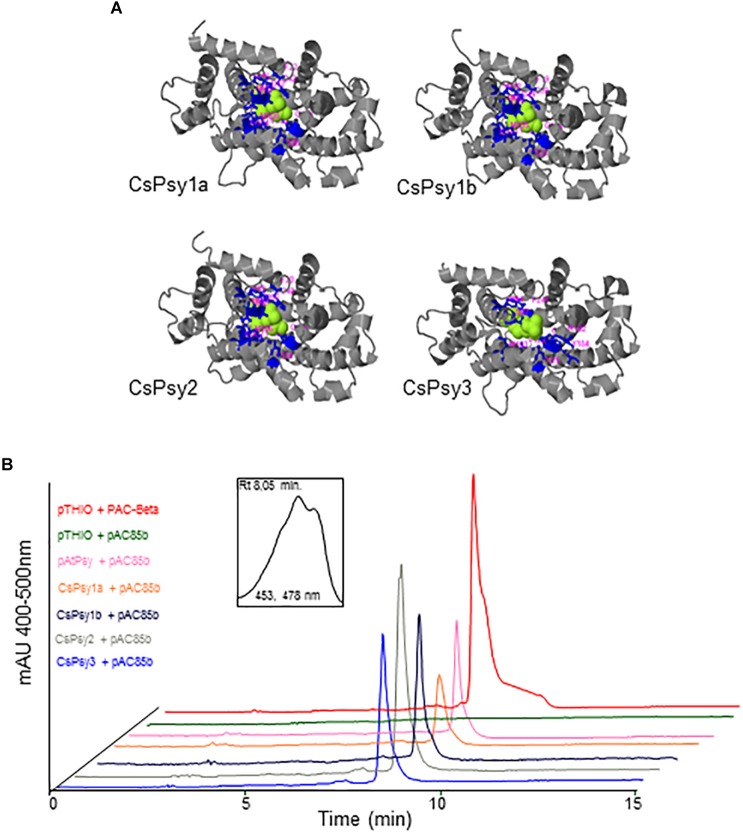
Functional complementation of the saffron PSY proteins. **(A)** Complex models of the CsPSY enzymes and their co-factor. **(B)** *Escherichia coli* cells harboring the pAC-85b vector were additionally transformed with saffron PSY constructs or empty vector. Cells carrying the pAtPSY vector confer accumulation of β-carotene and were used as a positive control. HPLC chromatograms for the extracted pigments are shown. The peak representing β-carotene was observed in cells with CsPSY1a, CsPSY1b, CsPSY2, and CsPSY3 constructs, but not in the empty vector. The inset shows the absorption spectrum of the β-carotene peak.

All four CsPSY enzymes showed very similar tridimensional structures, with the active site surrounded by the same amino acid residues (Figure 7A). To investigate the functionality of CsPSY1a, CsPSY1b, CsPSY2, and CsPSY3, the ORF of each corresponding gene, truncated to remove the signal peptide, was subcloned and expressed in *E. coli* cells containing the pAC-85b plasmid, which contains the *crtE*, *crtI*, *crtY* genes from *E. herbicola* Eho10, and will produce β-carotene in *E. coli* when complemented with a gene encoding a functional PSY enzyme. The expected product, β-carotene, confirmed by matching the spectra and column chromatography retention times, was produced in bacteria transformed with each CsPSY enzyme (Figure 7B). This indicates that *CsPSY1a*, *CsPSY1b*, *CsPSY2*, and *CsPSY3* cDNAs all encoded enzymes that were functional in the bacterial system (Figure 7B).

### Sequencing and Computational Analyses of Genomic Clones From the *CsPSY* Genes

All four *CsPSY* genes showed different 5′ UTR lengths (Supplementary Table S3) with *CsPSY1b* showing the longest one. Interestingly, an intron was present in the 5’UTR region of *CsPSY1b*. The presence of introns in the 5′ UTR has also been reported in tomato (Giorio et al., 2008), rice (Welsch et al., 2008), maize and Arabidopsis (Alvarez et al., 2016). Using 5′ UTR-specific primers for *CsPSY1b*, we identified two alternative splice variants for *CsPSY1b* that differ in the intron retention of the 5′ UTR (Supplementary Figure S3). We investigated whether the two identified transcripts behave differentially in distinct tissues and in response to light, and we found that in tissues in which *CsPSY1b* is highly expressed, such as corms, roots or leaves, the *CsPSY1b* transcript variant lacking the intron sequence was the only one detected (Supplementary Figure S4), while in those characterized by low *CsPSY1b* expression levels, both variants were detected but differed in their abundance (Supplementary Figure S4). We further analyzed the secondary structure of the *CsPSY1b* 5′ UTR with the intron sequence, and the prediction indicated the formation of a stable imperfect stem loop upstream of the ATG codon (Supplementary Figure S4), which is lost in the splice variant, since the intron removes most of the sequence participating in the formation of this structure (Supplementary Figure S4). In general, *PSY* genes are characterized by the presence of several intronic sequences (Li et al., 2008b), a finding also confirmed in the case of the coding region of the four *CsPSY* genes (Supplementary Table S3). *CsPSY1a* showed four exons, *CsPSY1b* showed three, and *CsPSY2* and *CsPSY3* four. Certain intron positions are conserved among the *CsPSYs*. For example, the five intron positions of the *Selaginella moellendorffii* and *Physcomitrella patens PSY* genes (233658 and Pp3c24_16390v3.1 from^15^) are conserved in five out of the six introns in the *Arabidopsis thaliana PSY* gene and in the three *PSY* genes from maize (Li et al., 2008a). In the *CsPSY1a* exons, one and two and four and five are fused; in the *CsPSY1b* exons three, four, five, and six are all fused together; in the *CsPSY2* exons one, two, and three are all fused together and in *CsPSY3*, the first four exons are all fused (Supplementary Figure S5).

## Discussion

The enzyme PSY has been shown to determine the rate of carotenoid accumulation in non-green plant tissues (Nisar et al., 2015), and in most plant species, it is represented as a small gene family, which reflects an ancient functional specialization of the PSY paralogs. We identified four genes encoding the PSY enzymes in saffron and designated them as *CsPSY1a*, *CsPSY1b*, *CsPSY2*, and *CsPSY3*. All four CsPSY enzymes were localized in plastids, but certain specific characteristics were observed. CsPSY1a showed a restricted location inside the plastid, while CsPSY1b, CsPSY2, and CsPSY3 were localized to chloroplasts in specific fixed speckles that were suggestive of plastoglobuli, since ZmPSY2 and ZmPSY3 are also located in plastoglobuli (Shumskaya et al., 2012).

### The Implication of CsPSY1 Genes in Leaf Carotenogenesis and Stress Responses

A phylogenetic tree shows that the saffron PSY proteins group within different categories (PSY1, PSY2, and PSY3) rather than with themselves, suggesting that the duplication and functional specialization of these genes preceded the separation of the major plant lineages (Li et al., 2008a). Two PSY1 enzymes were found in saffron, as well as in other plant species, such as tomato, which were probably generated by the *Solanum* whole-genome triplication The Tomato Genome Consortium (2012). There are two copies of *PSY1* encoding genes in the genome of *Malus domestica*, but there is no information on their expression profiles (Ampomah-Dwamena et al., 2015). In addition, six copies are present in *Brassica napus* (Lopez-Emparan et al., 2014), *Brassica rapa*, and *Brassica oleracea*, which were generated during the subgenome triplication event, and all the *PSY* genes in these three Brassica species showed overlapping redundancy (Cardenas et al., 2012). The phylogenetic tree showed that the two copies for PSY in saffron, CsPSY1a and CsPSY1b, did not group together, as was observed for tomato, *M. domestica* and *B. napus*. Interestingly, two copies of the PSY1 enzymes were also identified in the other autumn Crocus, *C. cartwrightianus* (section Crocus), but were not identified in two spring Crocus species, *C. sieberi* (Ahrazem et al., 2018) and *C. ancyrensis* (Ahrazem et al., 2015a), both in the Nudiscapus section, suggesting that the *CsPSY1* genes were probably products of tandem or segmental duplications after the separation of the Crocus and Nudiscapus sections. The *CsPSY1a* and *CsPSY1b* genes were mainly expressed in photosynthetic tissues but showed some differences at the level of expression. Carotenoids in photosynthetic tissues have different functions, including action as accessory pigments, the stabilization of the thylakoid membrane and the acceleration of photomorphogenesis (Shumskaya and Wurtzel, 2013). *CsPSY1a* expression was higher in white and yellow leaves, which are developing in plants that are below the soil surface, suggesting its involvement in the process of de-etiolation. When leaves emerge to the surface and grow, both the *CsPSY1a* and *CsPSY1b* transcripts increased along with the leaf blade, but the expression levels of *CsPSY1b* appeared to be more directly correlated with the distance of the leave tip from the base, since *CsPSY1b* is expressed at greater levels as the distance to the base of the leaf increases. Such expression patterns can be associated with the developmental stage of the chloroplast along the leaf and to the increasing number of plastoglobuli. In monocotyledonous leaves, the process of the transformation of proplastids into functional chloroplasts can be observed as a gradient along the leaf (Pogson et al., 2015). During this process, as a lipid reservoir, the plastoglobuli may assist in the rapid formation of thylakoid membranes in greening tissues, supplying lipid building blocks for membrane expansion, a process which may explain why plastoglobuli become rare and small during thylakoid formation but are more abundant and larger in the mature chloroplast (Rottet et al., 2015). In addition, *CsPSY1b* expression was associated with light stress, while *CsPSY1a* was unresponsive. The studies in the roots under different abiotic stress conditions also indicate that *CsPSY1b* appeared to be more involved in stress-associated responses than *CsPSY1a*. Interestingly, we found that the expression of *CsPSY1b* was regulated by alternative splicing within its 5′ UTRs, which generates two distinct mRNAs that differ solely in their 5′ UTRs length. The distribution of the different *CsPSY1b* transcripts was found to exhibit tissue specificity. Interestingly, the *CsPSY1b* variant resulting from the retention of a 536-nt intron in the 5′ UTR with a predicted stable stem loop structure appeared to be predominantly expressed in those tissues with global lower *CsPSY1b* expression levels. The 5′ UTR intron retention event in *PSY* has also been reported in Arabidopsis (Alvarez et al., 2016), where the identified stable stem loop due to intron retention appears to confer translational inhibition. In general, in other plant species, orthologs to *CsPSY1* showed that their expression levels are correlated with carotenoid biosynthesis in photosynthetic tissues, as is the case with carrot (Wang et al., 2014), cotton (Cai et al., 2014), and melon (Qin et al., 2011). In addition, the rice *PSY1* and *PSY2* are both photoregulated (Welsch et al., 2008), and the loss of photoregulation in the maize *PSY1* gene suggested that the ancestral Poaceae *PSY* was a photoregulated gene (Li et al., 2008b).

### The Implication of CsPSY2 in Crocins Biosynthesis

*CsPSY2* expression was prominent in the stigma tissue, and the expression profile followed the accumulation of saffron apocarotenoids along the development of the stigma (Moraga et al., 2009), suggesting that *CsPSY2* plays the main role in apocarotenoid biosynthesis in the stigma tissue, as has been found previously in other Crocus species (Ahrazem et al., 2015a, 2018). In addition, we found the presence of the *PSY2* orthologs in other plants that were associated with carotenoid and apocarotenoid accumulation in the chromoplast as in carrot, *Bixa orellana*, or *Eriobotrya japonica*. In the red-fleshed varieties of *E. japonica*, the gene encoding *EjPSY2A* was highly expressed in the fruit flesh and responsible for carotenoid accumulation in this tissue (Fu et al., 2014). In addition, the expression of the *B. orellana* ortholog is strongly associated with bixin biosynthesis in *B. orellana* fruits (Cardenas-Conejo et al., 2015).

The identification and analyses of *CsPSY2*, together with the previously identified chromoplast-specific lycopene-β-cyclase (*CYCB*) (Ahrazem et al., 2010) and β-carotene hydroxylase (*CHY*) (Castillo et al., 2005), indicate the presence of a specific carotenogenic pathway for apocarotenoid biosynthesis and accumulation in chromoplasts in the stigma tissue of saffron.

### Function of CsPSY3 in Strigolactone Biosynthesis in Roots

The CsPSY3 amino acid sequence was more closely related to the PSY3 sequences from dicotyledonous species than to the ones isolated from the Poaceae (Supplementary Figure S6). Orthologous sequences were identified in *Asparagus*, *Agave*, and *Allium* species. However, non-orthologous sequences encoding *CsPSY3* were identified in the genomes from other monocotyledonous species, such as *P. dactylifera*, *E. guineensis*, *C. nucifera*, *P. equestris*, *A. comosus, Z. maritima*, or *S. polyrhiza*. In these species, we did not find orthologs to ZmPSY3, suggesting the absence of PSY3 enzymes. In the Poaceae, PSY3 enzymes have been associated with the root carotenogenesis needed for drought and the salt stress-induced production of ABA (Li et al., 2008a). In dicotyledonous plants, PSY3 enzymes are predicted to function in the roots under stress conditions similarly to cereals. However, data on *Medicago truncatula* and tomato showed that *PSY3* is regulated by nutrient stress and mycorrhization, suggesting the involvement of PSY3 in apocarotenoid biosynthesis in roots (Favre et al., 2014; Walter et al., 2015). Interestingly, all the monocotyledonous species described above, which do not have a PSY3 ortholog, are characterized as having originated in tropical or subtropical regions or from aquatic habitats. Plants naturally occurring in such habitats are considered non-conducive to mycorrhizal fungi (Brundrett, 2009). In addition, in a recent study about root colonization by arbuscular mycorrhizal fungi, it was shown that this process is related to sites that feature continental climates with mild summers and a high availability of soil nitrogen (Soudzilovskaia et al., 2015). In addition, the genome of the dicotyledonous *Chenopodium quinoa* (PRJNA394242), *Dianthus caryophyllus* (PRJDB1491), *Beta vulgaris* (PRJNA268352), *Utricularia gibba*^16^, and *Raphanus sativus* (PRJNA344915) does not contain a ortholog of *PSY3*, and this can explain their inability to form arbuscular mycorrhiza symbioses (Urcelay et al., 2011; Rajkumar and Seema, 2012; Bravo et al., 2016), since this is also the case with *A. thaliana* and the other Brassicas (Delaux et al., 2014; Delaux, 2017). Interestingly, the authors of these studies used phylogenomic analyses on arbuscular mycorrhiza symbiosis in host and non-host plants and identified another gene, deoxyxylulose-5-phosphate synthase (DXS2), involved in carotenoid biosynthesis, as a potential symbiotic gene. In addition, *DXS2* is known to play a role during arbuscular mycorrhiza symbiosis (Floss et al., 2008). Saffron is strongly colonized by arbuscular mycorrhizae in the field (Kianmehr, 1981). *CsPSY3* was mainly expressed in the roots, and its expression was associated with stress responses, mycorrhizal colonization and the presence of orobanchol. However, among the *CsPSY* expressed in the root, *CsPSY3* was the unique *PSY* induced in the roots colonized by mycorrhizae. Therefore, as is the case with *M. truncatula* and tomato, the saffron PSY3 is a monocotyledonous protein with similar functionalities to the dicot enzymes, since it is associated with apocarotenoid biosynthesis during arbuscular mycorrhizal symbiosis (Walter et al., 2015; Stauder et al., 2018). The enzyme clearly differs from those identified in the Poaceae monocotyledonous plants, although it was also well induced under stress conditions.

In summary, four genes encoding PSY enzymes in saffron have evolved characteristic roles during carotenogenesis in different tissues. CsPSY1a and CsPSY1b were mainly associated with leaf carotenogenesis; CsPSY2 was related with chromoplast apocarotenogenesis, and finally CsPSY3 was associated with mycorrhizal-induced apocarotenogenesis.

## Data Availability

The datasets generated for this study can be found in GenBank, Accession number for MH124238, MH124237, MH124239, and MH124240.

## Author Contributions

LG-G and OA designed the research, analyzed the data, and wrote the manuscript. GD and AF performed the data integration analyses and eGFP location experiments. ÁR-M and JAP dissected the tissues, performed the RNA extraction and purification, and the expression analyses under the guidance of LG-G. CR, RV, and FM performed the strigolactone extraction and analyses. LG-G, RC, and OA performed the activity assays and genomic studies. All authors discussed the data and reviewed and commented on the manuscript.

## Conflict of Interest Statement

The authors declare that the research was conducted in the absence of any commercial or financial relationships that could be construed as a potential conflict of interest.

## Footnotes

1 www.clontech.com

2 http://www.clustal.org

3 http://www.megasoftware.net/mega.html

4 www.macrogen.com

5 http://www.ncbi.nlm.nih.gov

6 http://expasy.hcuge.ch/sprot/prosite.html

7 http://www.cbs.dtu.dk/services/SignalP

8 http://www.cbs.dtu.dk/services/DeepLoc/

9 http://www.cbs.dtu.dk/services/TMHMM/

10 http://www.isb-sib.ch

11 http://opm.phar.umich.edu/server.php

12 www.thermofisher.com

13 http://www.cbs.dtu.dk/services/

14 http://www.cbs.dtu.dk/services/TMHMM/

15 https://phytozome.jgi.doe.gov/

16 http://genomevolution.org/CoGe/
