# Supplementary material for: The Specialized Roles in Carotenogenesis and Apocarotenogenesis of the Phytoene Synthase Gene Family in Saffron
Source: Front Plant Sci. 2019 Mar 4;10:249. doi: 10.3389/fpls.2019.00249 (PMC6409354; doi:10.3389/fpls.2019.00249)
Supplement: Supplementary file 1 [file Data_Sheet_1.PDF]

Supplementary Figure S1

a)

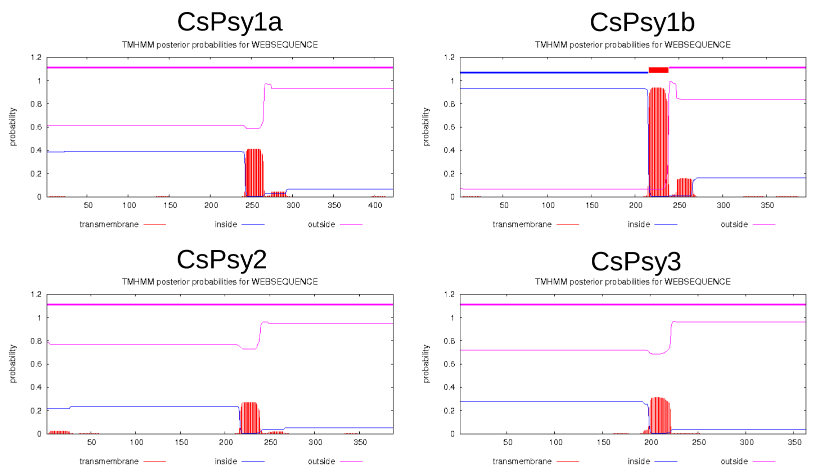

b)

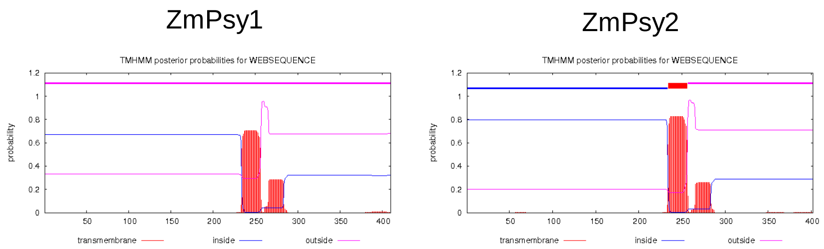

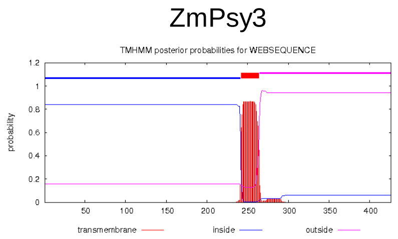

Location in plastids:

- ZmPSY1: peripherally associated with membranes.
- ZmPSY2: plastoglobuli
- ZmPSY3: plastoglobuli

c)

Name	Len	cTP	mTP	SP	other	Loc	RC
CsPSY1a	423	0.844	0.058	0.168	0.671	C	3
CsPSY1b	395	0.839	0.075	0.021	0.103	C	2
CsPSY2	388	0.818	0.057	0.011	0.342	C	3
CsPSY3	363	0.760	0.042	0.023	0.326	C	3

Supplementary Figure S1. (a) Putative hydrophobic profiles of different CsPSY enzymes, evaluated by the TMHMM Server v. 2.0. (b) Putative hydrophobic profiles of different ZmsPSY enzymes, evaluated by the TMHMM Server v. 2.0 and their location in plastids according to Shumskaya et al., 2012. (c) Prediction of the subcellular location of CsPSY proteins using the TargetP 1.1 Server.

Supplementary Figure S2

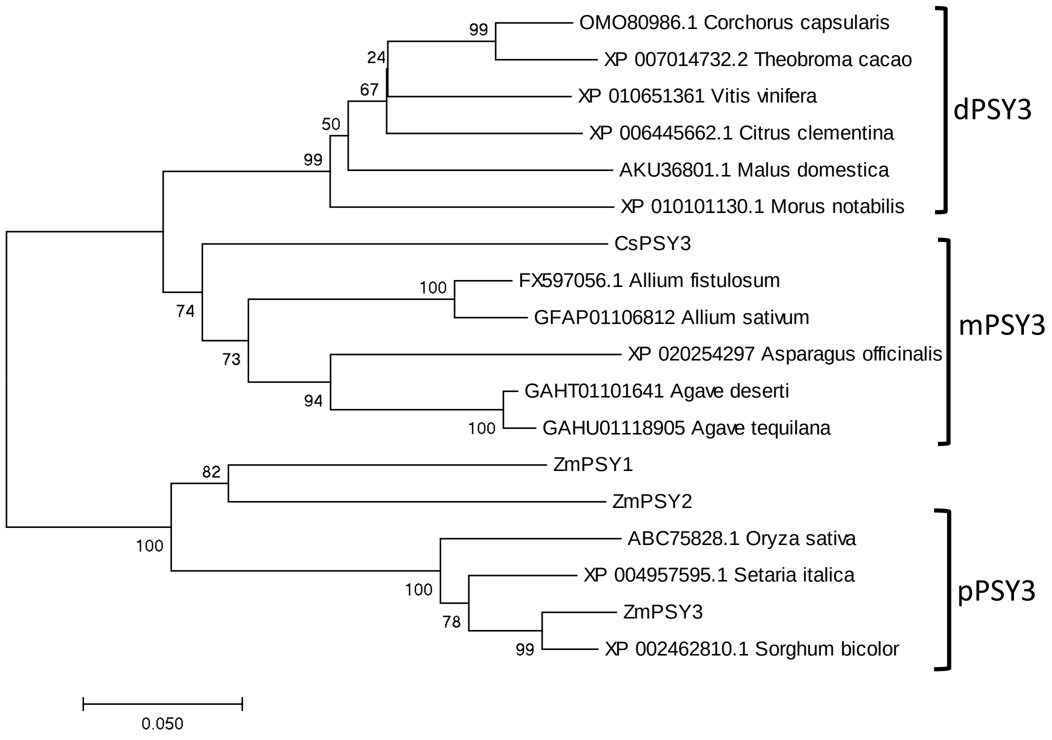

Supplementary Figure S2. Phylogenetic tree (neighbor-joining) of PSY3 enzymes from monocots (excluding the Poaceae sequences: mPSY3), Poaceae sequences (pPSY3) and dicots (dPSY3). The length of branch expresses evolutionary distance, with its scale being 0.05.

CsPSY1b 5’UTR

AGAAATAATAGTAATAAAAAATTTGCATTATTTTATCTTCTAAGAAAATGATTGGTGATTTTTTTTTCTC
TTTGTTTTTTGGGAGAGAGGTGGTTGATTCTATACCTAGCATGCCATGGTTCTCATGCTAGGAATTCAAT
GGTATATAATGGTGTGGTAGTGGTTGATAGTCATTCATCACATGCTATTTGGAAACACAAAGATAAGAGA
TACCCAATTCCACCACTTAACTTGCCCCTCTCTTCTTCTTCTTCTTCTTCTTCTTCTTCAAAGTTCATTC
ACTTCTTTTGGGGGCATCTTGTCATTATTGAATACTGAAGGTAACATACTATTCATATCATATTCCTTTT
TGGTATCTTAGCATTGTTCTTTTTTTAATGTATGTCCATTTTCATCCAAGTGGCTCTACTTTTTCTCTTGA
ATGCTAATAACACCATACTATTCCTAGGATAATTTTGGCATGTGGGTCTTGGTTGTTATGATTATTGTCT
AGCTTTTGTTGGTATATCTCCACTTCTTCTCTTTTTGTTTTTTGTAAGAGTAGAAAAAAAAGGAACCTCC
TTTTTTCTCTCCCTTCATCATGTCATTGTTGATTGGATACTAAAGATGACACTTTTCCTCTTTTCTTGGT
ATGGATTTCACCAAGTCATCTAAGTTTTTCAATATTGAGCAATGAATCTGAATATCTGTTATAAATACCC
ATCAGAAGTTCCTCTCCTTTGTGTTTCAATCATGTCACTGTTGATTGGATACTGAAAAGTAACATCTTGA
AGAATTTTAGCATATGGGTTTGTGATAGTTTATGATTACCTTGTGATTCATGTAGTTAATTTACTTACAA
TACTCTACTTCTTTAGAGTTGTAATCACAAGGATTTGAAACTCTGCTGGA**ATG**

Supplementary Figure S3. *CsPSY1b* 5’UTRs with the intronic sequence in white background, and the start codón in red.

Supplementary Figure S4

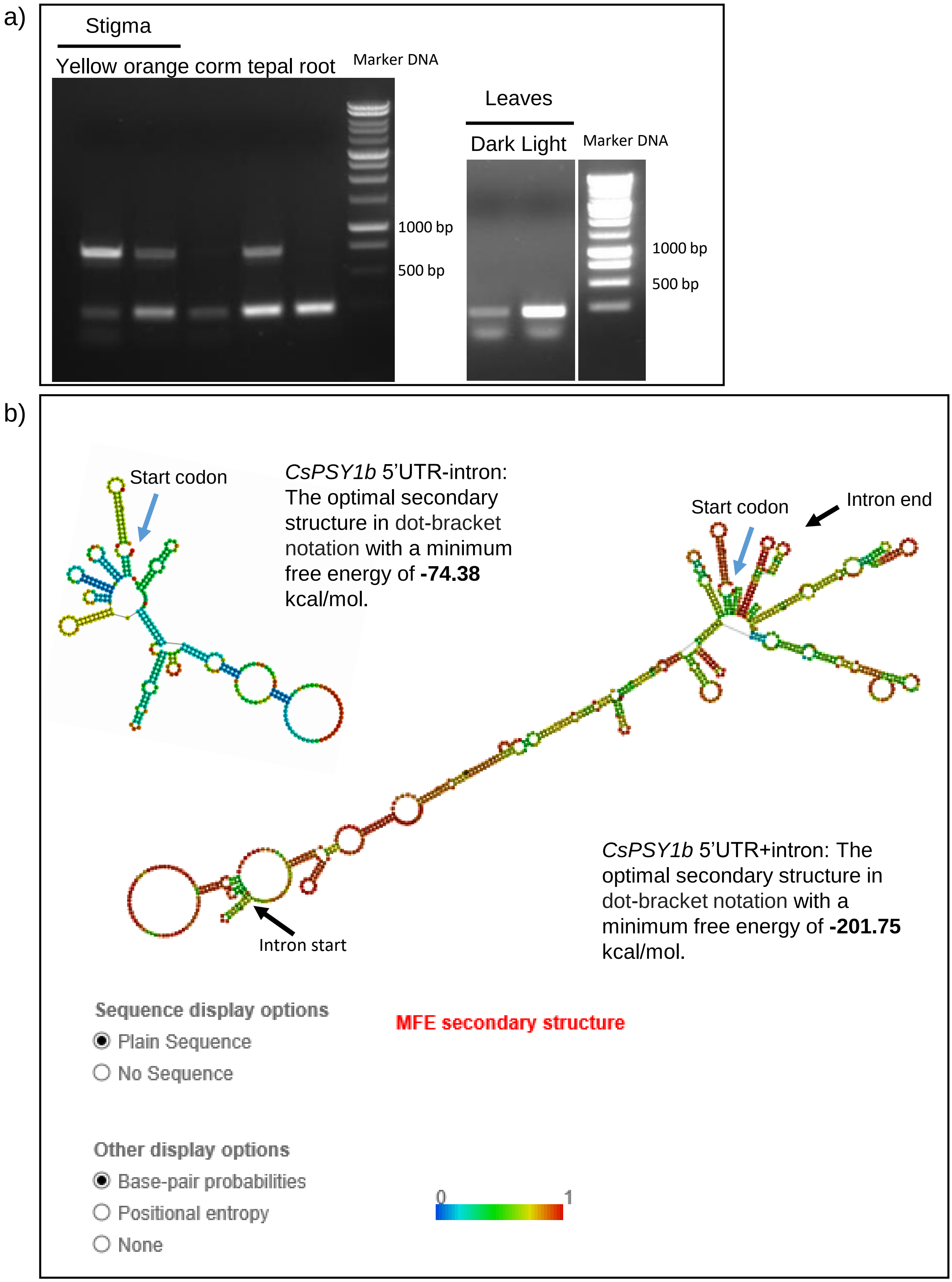

Supplementary Figure S4. Selective expression of alternative CsPSY1b variants. (a) Agarose gel showing the results of RT-PCR over CsPSY1a 5′UTR. (b) Structures of PSY 5′UTRs with or without the intron sequence performed with RNAalifold .

Supplementary Figure S5

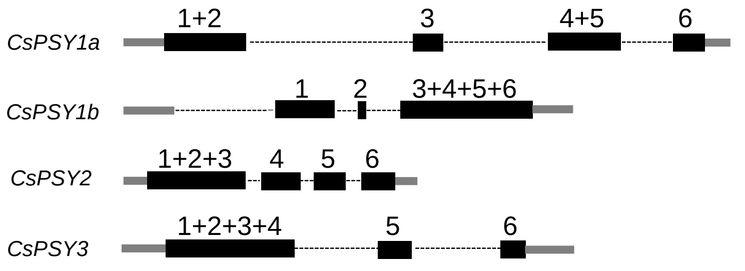

Supplementary Figure S5. Exon/intron structure of genomic *CsPSY* genes. Identified exons are shown as solid black blocks. Introns are shown as dotted lines and untranslated regions are shown as grey lines.

Supplementary Figure S6

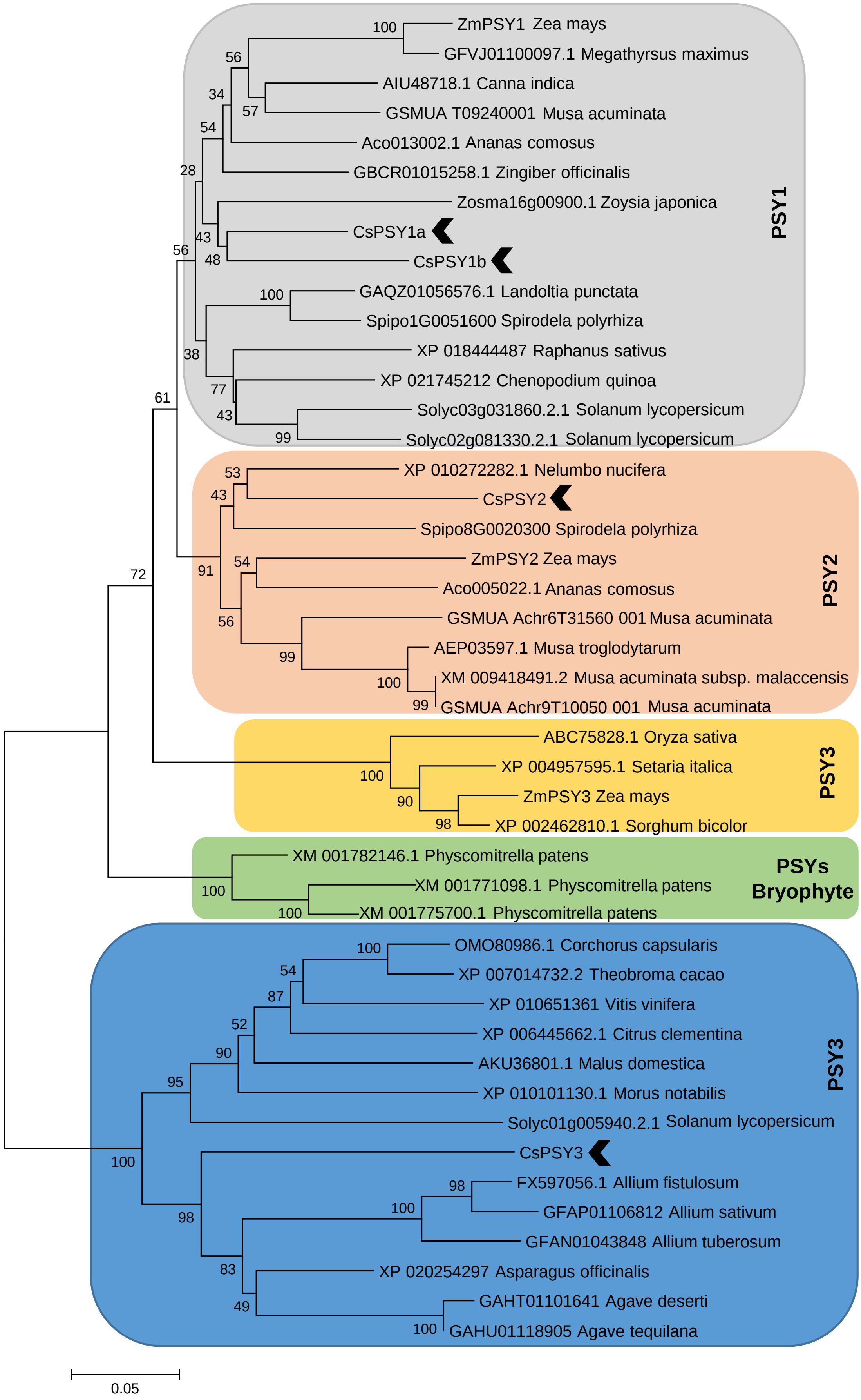

Supplementary Figure S6. Phylogenetic tree (neighbor-joining) of PSY enzymes. Indicated by a black arrow are CsPSY enzymes isolated in this study. Numbers associated with the branches are the neighbor-joining bootstrap values. The length of branch expresses evolutionary distance, with its scale being 0.05.

Supplemental Table S1. Oligonucleotide sequences used in this study.

	Forward 5'-3'	Reverse 5'-3'
For cDNAS isolation	1-GARTAYGCIAARACITTYTAYYT<br>2-ATHTGGGCIATNTAYGTITGGTG	TCRTCYTGIGGIAGRTADATYCT
For RACE-PCR		
CsPSY-1a	GTGTTCCGGTGATGGGTATCG	CTTGAATGGCTGAATGTCAAC
CsPSY-1b	TGCACCAGACTCTCAAGCCAC	GTCCATCTACAAGCTCATCTGT
CsPSY-2	TCAGCTTACCAACATACTAAGA	CATCCACAAGTTCATCGGTTC
CsPSY-3	AGGCATGCAACTCACCAACATC	TCTTGCCATCGATCAAGGACA
For full-length cDNA and gDNA isolation		
CsPSY-1a	ATGGCAATCGCATTACTTCGAGT	TCATCCTTTCAAGAGGCTCGACT
CsPSY-1b	ATGGCAACTCAAATGATGAGGGT	CTAGGCATTACCAGGTTGGCTAC
CsPSY-2	ATGTCTGGTTCTGTTGTTTTGTTG	TCAGACTTTTGAAGGACCAAATG
CsPSY-3	ATGAGTACCTCCATTTTACCAAAT	AGCTTTAGCAAATCCCCAAGGCA
For expression analyses		
CsPSY-1a	TTCGCCCATAGAATTTCCAA	TAAACCCTCTGCTCCGAAGA
CsPSY-1b	GTTGTCTCACCGGTGGAAGT	TCACCAATTCGAGGTTGACA
CsPSY-2	TTTCGGTCACCACCGATAAG	TCATGAGCAATGTTCCCAAG
CsPSY-3	CACTATCCAGCTTCCCTCCA	CCACGACACCTCCTATAGCC
For activity assays		
CsPSY-1a	CGCCCTTGGCGAATTCGAAGGAGACCTTAG AGGA	TACCCTCGAGGAATTCTCATCCTTTCAAG AGGCTC
CsPSY-1b	CGCCCTTGGCGAATTCGCGAACCCGGTGAA AGAT	TACCCTCGAGGAATTCTCATTACCAGGTT GGCTAC
CsPSY-2	CGCCCTTGGCGAATTCCATCACTTCACTGG AAGG	TACCCTCGAGGAATTCTCAGACTTTTGAA GGACCA
CsPSY-3	CGCCCTTGGCGAATTCGTGAACGAGGTCGT GGTA	TACCCTCGAGGAATTCTCAAGCAAATCCC CAAGGCA
For GFP fusion		
CsPSY-1a	GGACTCTTGACCATGGATGGCAATCGCATT ACTTCGAGT	GTCAGATCTACCATGGTCCTTTCAAGAGG CTCGACTGCC
CsPSY-1b	GGACTCTTGACCATGGATGGCAACTCAAAT GATGAGGGT	GTCAGATCTACCATGCGGGCATTACCTTG GCTACCTTGC
CsPSY-2	GGACTCTTGACCATGATGTCTGGTTCTGTT GTTTTGTTGATTTCA	GTCAGATCTACCATGCCGACTTTTGAAGG ACCAGCAAGAGC
CsPSY-3	GGACTCTTGACCATGATGAGTACCTCCATT TTACCAAATCTCTCTATG	GTCAGATCTACCATGAGCTTTAGCAAATC CCCAAGGC
For 5'UTR CsPSY1b analyses	AGTGGTTGATAGTCATTCATC	GACAACCCTCATCATTTGAGT

Supplemental Table S2. Characteristics of the PSY proteins from saffron.

Name	Accession number	cDNA size (bp)	Protein size (aa)	Mw (KDa)	PI
**CsPSY1a**	MH124237	1272	423	47.79	8.66
**CsPSY1b**	MH124238	1187	395	44.24	8.86
**CsPSY2**	MH124239	1209	388	43.97	9.92
**CsPSY3**	MH124240	1098	366	42.53	6.28

Supplemental Table S3. Characteristics of the saffron PSY genomic clones

	Intron number	Intron size	5'UTR	3'UTR
**CsPSY1a**	3	958/619/316	307	149
**CsPSY1b**	3	536 (5'UTR)/143/256	890	235
**CsPSY2**	3	73/90/89	167/107	103
**CsPSY3**	2	524/539	264	293

Supplemental Table S4. Mass pair (m/z) and compound-dependent parameters of the standards.
† C.E.: Collision Energy

Compound name	t_R(min)	Quantifier ion (m/z)			Qualifier ion (m/z)		
		Q1 Precursor ion	Q3 Product ion	C.E † (eV)	Q1 Precursor ion	Q3 Product ion	C.E.† (eV)
**(±)-GR24 (IS)**	3.47	321	224	11	-	-	-
**Orobanchol**	3.67	347	233	12	347	329	6
